# Intrathecal Morphine Use Improves Postoperative Analgesia and Reduces Opioid Consumption in Patients Undergoing Total Knee Arthroplasty Under Spinal Anesthesia: A Retrospective Study

**DOI:** 10.7759/cureus.43039

**Published:** 2023-08-06

**Authors:** Promil Kukreja, Charlotte Streetzel, Roland T Short, Scott E Mabry, Joel Feinstein, Kathy Brazeel, Diana Cerice, Luanne Chapman, Hari Kalagara

**Affiliations:** 1 Anesthesiology and Perioperative Medicine, University of Alabama at Birmingham (UAB), Birmingham, USA; 2 Orthopaedics, University of Alabama at Birmingham (UAB), Birmingham, USA; 3 Anesthesiology and Perioperative Medicine, Mayo Clinic, Jacksonville, USA

**Keywords:** total knee arthroplasty, post-operative analgesia, spinal anesthesia, adductor canal block, intrathecal morphine

## Abstract

Background

Intrathecal morphine (ITM) provides effective postoperative analgesia for patients undergoing total knee arthroplasty (TKA) under spinal anesthesia (SA). The management of pain in patients undergoing TKA has remained a challenge for anesthesiologists, as no single regional anesthesia technique is adequate with regard to balancing effective analgesia with minimal side effects. Severe postoperative pain following TKA has been shown to negatively impact patient outcomes and mortality. This study is aimed to describe the effect of intrathecal morphine in patients undergoing total knee arthroplasty.

Methods

This was a retrospective, descriptive, and single-center study conducted on patients undergoing total knee arthroplasty from June 1, 2022, to June 1, 2023. The sample size consisted of 50 patients who were 18 years and older, American Society of Anesthesiology (ASA) class 1-3, and patients who had received either 150 mcg (experimental) or no ITM dose under spinal anesthesia. Oral morphine requirement (OME) and visual analog pain scale (VAS) were used to assess pain in the first 24 hours after surgery.

Results

The experimental group had significantly lower OME usage in the post-anesthesia care unit (PACU) (p < 0.001) and at six hours (p = 0.040) postoperatively. At 12 hours and 24 hours postoperatively, the two groups had similar OME use (p > 0.20, for both). The experimental group had significantly less total OME use over the first 24-hour postoperative period. The experimental group had significantly lower pain scores in the PACU (p < 0.001) and at six hours postop (p = 0.002); there were no significant differences between groups at 12- and 24-hours postop. The ambulation distance was clinically significant and better in the ITM group but was not statistically significant (p = 0.080). There was no difference between groups in the incidence of postoperative nausea and vomiting (PONV).

Conclusion

The careful use of ITM with the optimal dose offers an effective addition to regional anesthesia for improved analgesia with minimal side effects. The 150 mcg ITM dose provided good analgesic effects with longer duration and was not associated with respiratory depression.

## Introduction

As the population in the United States and other developed countries continues to experience increases in life expectancy and body mass index (BMI), osteoarthritis will continue to be a prevalent and life-altering disease [1-3]. In the United States, total knee arthroplasty (TKA) remains one of the most common and most painful orthopedic procedures [2]. Approximately 600,00 TKA procedures are performed each year and estimates indicate greater than three million people will undergo TKA by the year 2030 [4-6]. According to a randomized controlled trial published in 2020, approximately 20% of patients undergoing TKA under the current anesthetic practices are dissatisfied, and the majority of this dissatisfaction is related to severe postoperative pain [2,5]. Despite the large number of TKA procedures being performed, postoperative pain continues to be a major concern and a “gold standard” anesthetic pain management plan does not currently exist [1,2,7-9]. In this study, we investigated the impact of adding ITM to our existing pain management regimen for primary TKA.

Early ambulation is a crucial endpoint following TKA, as it is well known to contribute to improved functional outcomes, reduced hospital length of stay, and decreased risk of developing deep vein thrombosis (DVT) and pulmonary embolism (PE) [4-5,7]. As such, adequate pain control is required to facilitate early ambulation. Despite the increasing number of TKA surgeries performed and the advancements of regional anesthetic techniques, there is yet to be a clear superior mode of postoperative pain control for total knee replacement [10].

According to Memtsoudis et al., in a consensus statement published in 2021, by the International Consensus on Anesthesia-Related Outcomes after Surgery (ICAROS), peripheral nerve blockade (PNB) may be the optimal solution for effective pain control, a minimized side-effect profile, and a significantly lower odds ratio of adverse outcomes compared with total joint replacements performed without PNB [6]. Of note, the availability of facility resources as well as physicians trained in performing PNB procedures was a limiting factor recognized in their analysis, and the use of intrathecal morphine as an analgesic method for total joint replacement was not included in this comparison.

The use of intrathecal morphine for total joint replacement shows conflicting data. In 2022, a meta-analysis by the Procedure Specific Postoperative Pain Management (PROSPECT) working group concluded the use of intrathecal morphine for total knee replacement to be better than placebo but equivalent to single-shot and continuous nerve block catheters [2]. They found that the use of ITM also led to reduced postoperative opioid consumption in the immediate (6-12 hr) period, but all studies also demonstrated an increased risk of pruritis and decreased patient satisfaction [2-3]. For these reasons, they recommended the use of ITM only in situations when RA could not be performed. However, several other studies indicate that due to the hydrophilic properties of morphine, its prolonged duration of action within the intrathecal space makes it an appropriate option for addressing postoperative pain following TKA [7]. In an attempt to maximize pain control and minimize side effects related to the use of ITM for TKA, several dose-response studies have been performed. One study including 66 patients receiving ITM found that compared to those receiving 100 mcg of ITM, those who received 150 mcg of ITM had superior pain scores and found no difference in rates of respiratory depression in the 150 mcg ITM group [7]. A systematic review published in 2021 by Wang et al. indicates that the efficacy of intrathecal morphine (ITM) for total joint replacement has yet to be fully established [3]. Furthermore, despite the various advantages of ITM, including the efficiency of administration and cost-effectiveness, there are concerns regarding the safety and side-effect profile of ITM, including pruritus, respiratory depression, urinary retention, and PONV [3,5-7,11-14].

To date, no studies have formally compared continuous catheter adductor canal blocks (CCACB) versus single-shot adductor canal blocks (SSACB) plus intrathecal morphine (ITM) for patients undergoing primary knee arthroplasty. In developing a new Enhanced Recovery Program (ERP) for our TKA patient population, our department changed from providing CCACB plus spinal anesthetic (SA) alone to providing SSACB plus SA with ITM. This practice change presented an opportunity to retrospectively study our pre- and post-implementation patient populations. We hypothesized that the combination of SSACB plus SA with ITM would be non-inferior to that of CCACB plus SA alone in regard to postoperative pain, side effects, and patient satisfaction while simultaneously improving practice efficiency. To test our hypothesis we conducted this retrospective study of our TKA patient population, analyzing pre- and post-implementation data for ITM.

## Materials and methods

In this, our institutional review board (IRB)-approved retrospective study, we selected two groups of patients who had undergone primary total knee replacement. One group had undergone primary total knee replacement with continuous adductor canal block (CCACB) under spinal anesthesia without intrathecal morphine (control group). The other group had undergone primary total knee replacement with a single-dose adductor canal block (SSACB) under spinal anesthesia with the addition of intrathecal morphine (experimental group).

The adductor canal block (ACB) was performed preoperatively in our regional block area. Patients were consented for the block and the site confirmed during time-out for the block. Both groups received 50-100 mcg of intravenous fentanyl for sedation during the block. The patient was positioned in the supine position, and a linear high-frequency probe was used to identify the target. Both groups received a bolus of 25 ml of 0.5% ropivacaine for the adductor canal block. The nerve block catheter was placed in the CCACB group after the bolus. Both groups received intraoperative periarticular infiltration of the knee joint by a surgeon. The CCACB group received a continuous infusion of 0.2% ropivacaine at the rate of 6 ml/hour in the postoperative period. We utilized a random numbers generator to select 25 patients from the TKA patient pool from each group. Inclusion criteria consisted of any adult patient 18 years or older, who underwent primary total knee arthroplasty. Rescue pain medication was given orally either as oxycodone or hydrocodone and oral morphine equivalent (OME) was calculated for comparison between the two groups. Exclusion criteria included patients with a past medical history of chronic pain or chronic opioid use greater than 30 days and patients undergoing any surgeries other than primary TKA.

Data were summarized using means and standard errors (SE) for continuous outcomes or counts and percentages for categorical outcomes. T-tests and chi-square tests, as appropriate, were used to compare the groups on the study endpoints. A p-value < 0.05 was considered statistically significant. SAS version 9.4 (SAS Institute Inc., Cary, NC) was used to conduct all statistical analyses. Data are expressed as means and standard errors (continuous variables) or counts and percentages (categorical variables). The Wilcoxon rank-sum test was used to compare oral morphine equivalent (OME), pain scores, and ambulation distance.

## Results

Data were available for 50 subjects. Twenty-five subjects received intrathecal morphine (experimental group) and 25 received standard of care (control). Demographic information for the subjects is shown in Table 1.

**Table 1:** Demographic information by group * p-value from the two-sample t-test (age) or chi-square test (race/ethnicity, sex)

Characteristic	Control (n = 25)	Experimental (n = 25)
Age (years), mean (SE)	67.28 (1.93)	60.72 (2.32); P * 0.035
Race/Ethnicity, N (%)		P* 0.194
African American	7 (28.00%)	14 (56.00%)
American Indian	1 (4.00%)	0 (0.00%)
Hispanic/Latino	1 (4.00%)	1 (4.00%)
White	16 (64.00%)	10 (40.00%)
Sex, N (%)		P* 0.306
Female	18 (72.00%)	21 (84.00%)
Male	7 (28.00%)	4 (16.00%)

The two groups are compared on the study endpoints in Table 2, which include oral morphine equivalents (OME) and pain scores at various time points, use of anti-emetic medications, ambulation distance at 24 hours, and hospital length of stay.

**Table 2:** Study outcomes by group OME = oral morphine equivalent; PACU = post-anesthesia care unit * p-value from two-sample t-test (OME, pain, ambulation, length of stay) or chi-square test (use of antiemetic)

Outcome	Control (n = 25)	Experimental (n = 25)	P*
OME, mean (SE)			
PACU	34.26 (6.49)	1.50 (1.06)	< 0.001
6 hours	10.54 (2.76)	4.10 (1.30)	0.040
12 hours	11.19 (2.33)	7.80 (1.42)	0.220
24 hours	22.38 (3.67)	27.99 (5.21)	0.383
24 hours (cumulative)	77.21 (10.28)	42.59 (7.33)	0.009
Use of antiemetic, N (%)	8 (32.0%)	7 (28.0%)	0.999
Pain score, mean (SE)			
PACU	5.79 (0.73)	0.16 (0.16)	< 0.001
6 hours	4.72 (0.67)	1.80 (0.59)	0.002
12 hours	4.00 (0.77)	3.52 (0.69)	0.645
24 hours	5.43 (0.71)	6.00 (0.76)	0.591
Ambulation distance at 24 hours (feet), mean (SE)	67.56 (12.91)	98.88 (11.82)	0.080
Length of stay (hours), mean (SE)	37.96 (5.42)	33.20 (3.45)	0.462

**Figure 1:**
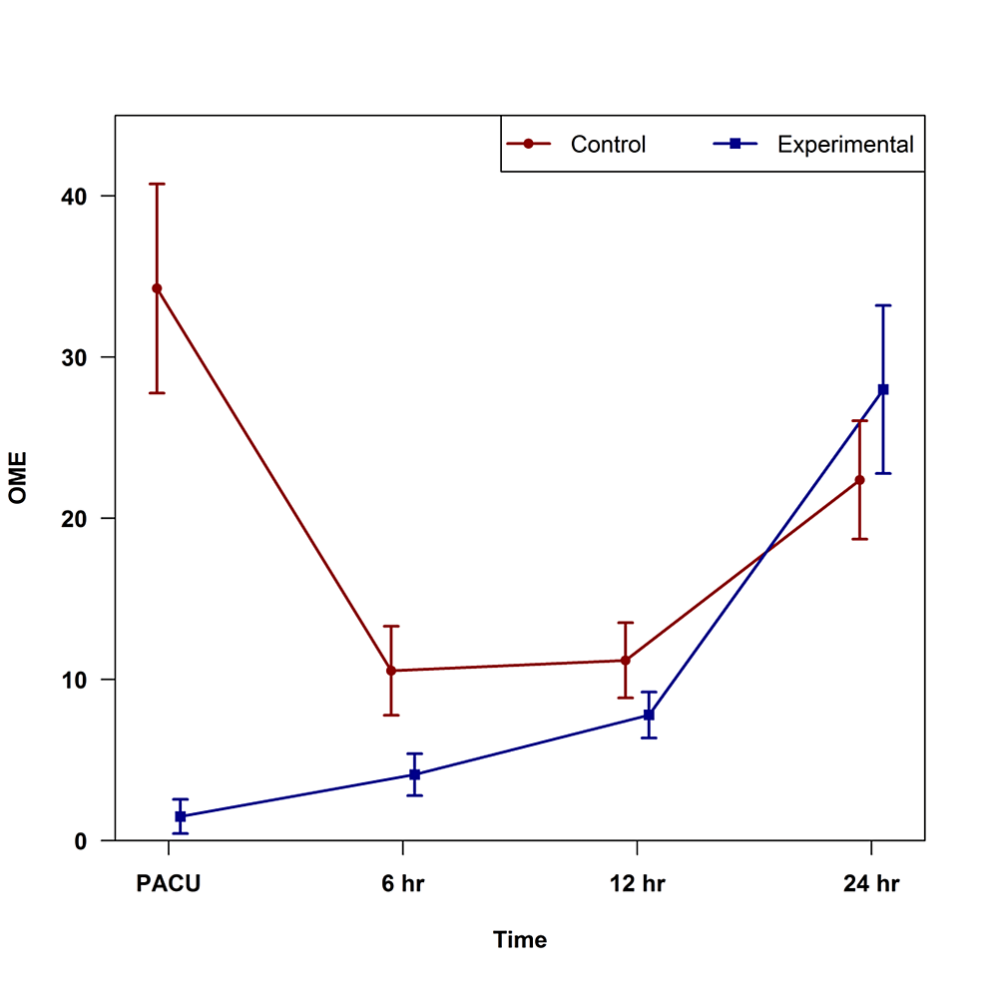
Oral morphine equivalent (OME) use over time by the group in the postoperative period

**Figure 2:**
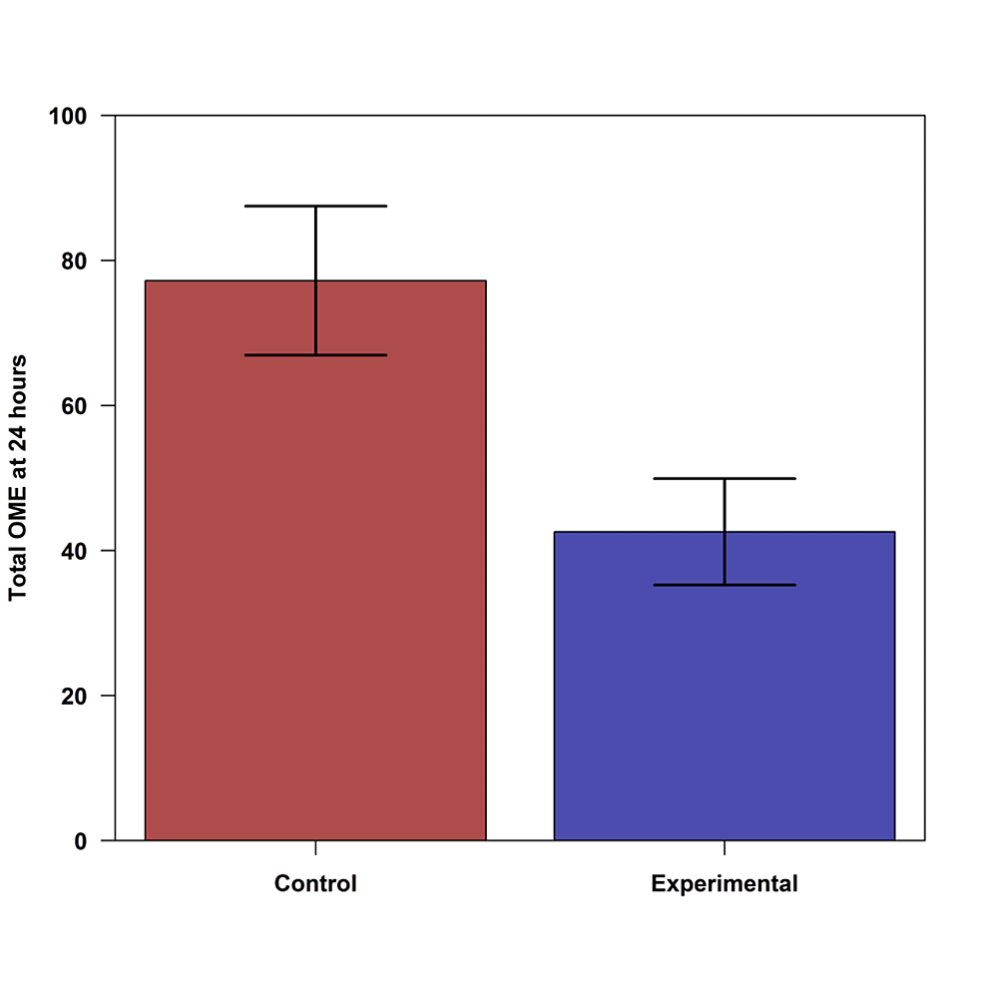
Cumulative oral morphine equivalent (OME) use at 24 hours by group

The experimental group had significantly lower OME usage in the post-anesthesia care unit (PACU) (p < 0.001) and at six hours (p = 0.040) postoperatively (Figure 1). At 12 hours and 24 hours postoperatively, the two groups had similar OME use (p > 0.20, for both). The experimental group had significantly less total OME use over the first 24-hour postoperative period (p = 0.009, Figure 2).

**Figure 3:**
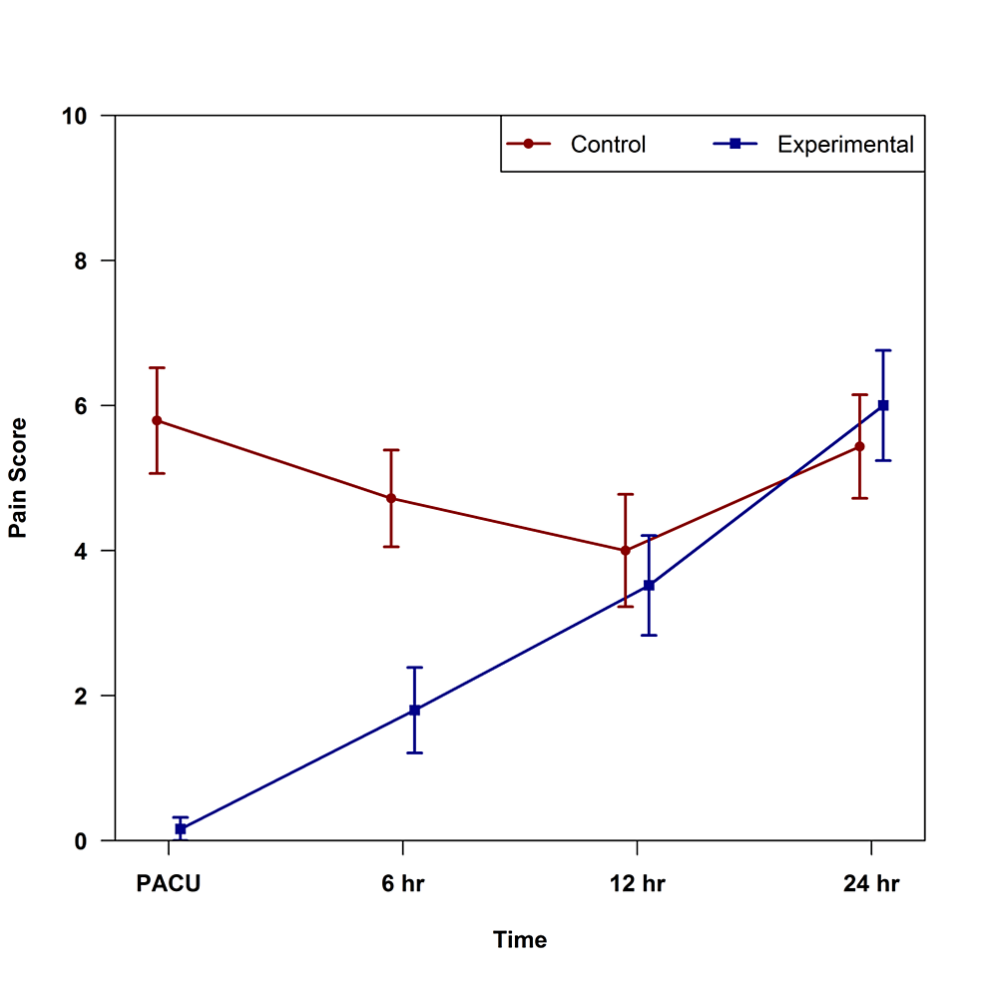
Pain scores over time by group in the postoperative period

**Figure 4:**
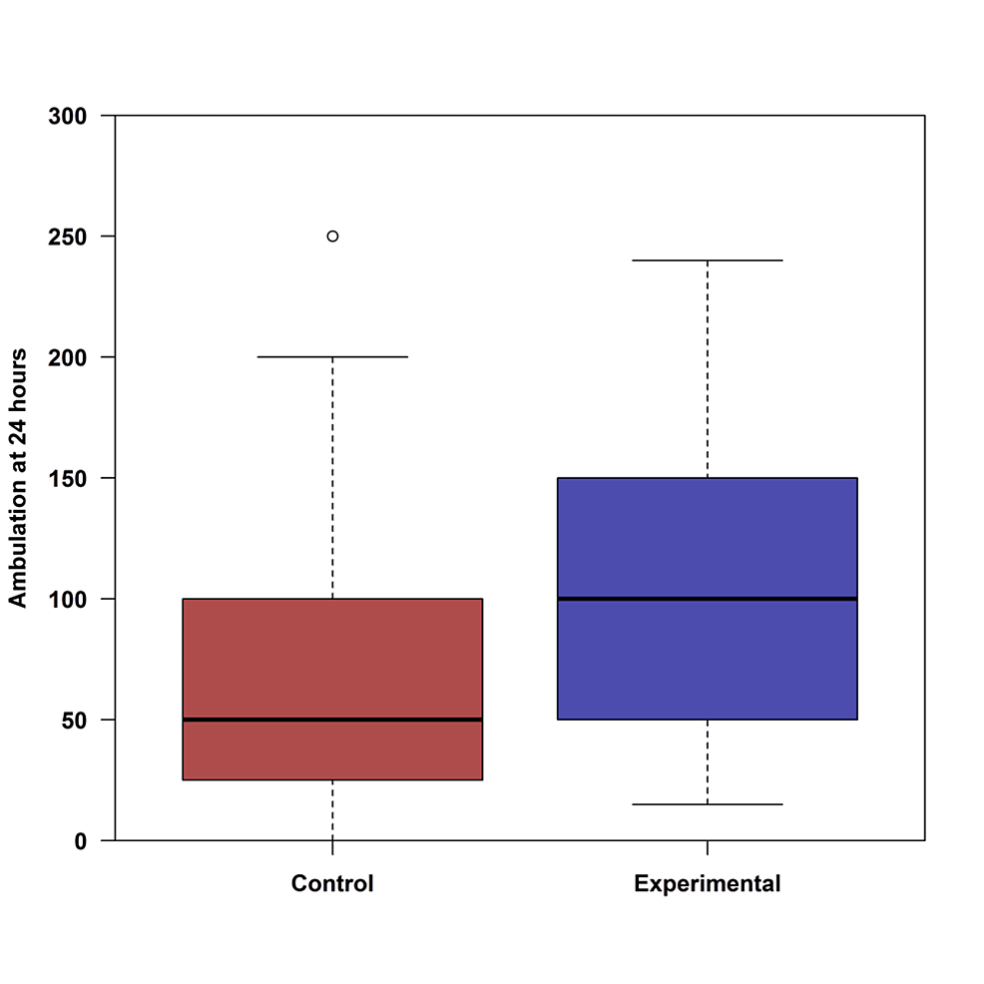
Ambulation distance in feet at 24 hours by group in the postoperative period

The experimental group had significantly lower pain scores in the PACU (p < 0.001) and at six hours postop (p = 0.002); there were no significant differences between groups at 12- and 24-hours postop (Figure 3). The ambulation distance for the two groups is shown in Figure 4; the ambulation distance was clinically significant and better in the ITM group but not statistically significant (p = 0.080).

There was no difference in the use of antiemetic medications between the two groups. We did not find any incidence of respiratory depression, pruritus, and the use of naloxone in either group.

## Discussion

This retrospective study demonstrates that compared to a CCACB plus SA alone, an SSACB plus SA with ITM provides effective analgesia after primary TKA. The decision to use a dose of 150 mcg of morphine for our intrathecal injection was based on several studies that suggest this dose provides maximal analgesia and minimal side effects [4].

For our primary efficacy outcome, our findings suggest that ITM provides both clinically meaningful and statistically significant analgesia in PACU at 6-12 h when compared to control. We used oral morphine equivalent (OME) as a more objective measure of analgesia efficacy. The effect was consistent with lower pain scores in the ITM group at earlier time points. The duration of the effect of ITM is estimated to be up to 16 h [14]. This is consistent with our study results where we did not find any statistically significant differences in analgesic outcomes at 24 h.

We also demonstrated that postoperative analgesia was more effective in the ITM group even in the presence of a regional anesthesia block when compared to the control group. Both groups received intraoperative periarticular infiltration of the knee with the same volume and concentration of local anesthetic. The superior analgesia provided by ITM questions the utility of continuous adductor canal block compared to single shot block. At our institute, most of our primary TKA patients are going home the day after surgery, and early ambulation is encouraged for better outcomes. The ITM group has shown increased ambulation distance when compared to the control group. This may be attributed to better pain control in the ITM group with less opioid consumption in the first 24 hours.

The incidence of PONV, pruritus, respiratory depression, and urinary retention was similar between groups; however, our study was not sufficiently powered to determine whether this finding was statistically significant. Intrathecal morphine has been shown to be associated with an increased risk of PONV, pruritus, and urinary retention, but without any impact on the duration of hospital stay [15]. Although a dose above 100 mcg statistically increased the rate of PONV according to a meta-analysis, we did not find any increased risk of PONV with 150 mcg of ITM when compared to the control. Of note, none of the patients in this retrospective study received dexamethasone, which has been reported to decrease PONV secondary to ITM use from 54% to 22% [16]. We also did not find any incidence of pruritus, urinary retention, or the use of naloxone for respiratory depression.

Because of concerns for sedation, hypoxia, and respiratory depression guidelines for the practice of neuraxial opioids have recommended that patients be continuously monitored for 24h after receiving ITM [17]. Respiratory depression has been reported with a high ITM dose of 250 mcg, but recent evidence supports our finding of the absence of respiratory depression with doses at or below 150 mcg [18]. The ITM dose of 150 mcg did not increase the incidence of sleep apnea or respiratory depression even in older patients undergoing hip arthroplasty [19].

There are some limitations to this case series such as small sample size, retrospective design, and publication bias [20]. The sample size in our study is too small to find any statistically significant differences for rare adverse outcomes like respiratory depression and urinary retention. These adverse effects can limit enhanced recovery for same-day arthroplasty, hence future studies with a big sample size are warranted to confirm this finding. Some results like ambulation distance were clinically significant but did not reach statistical significance due to the small sample size. Most of the patients were discharged postoperative day 1, so we could not collect consistent data past 24h of surgery. The long-term benefits of ITM could not be assessed due to the retrospective study design. The small sample size was intentional, as this study was also a part of the quality improvement initiative.

## Conclusions

There is strong evidence that intrathecal morphine provided effective analgesia after lower extremity arthroplasty. Our study concludes that ITM of 150 mcg for total knee arthroplasty under spinal anesthesia provided improved postoperative analgesia with reduced opioid consumption. Our study warrants no more concerns of PONV, pruritus, or respiratory depression with this dose of ITM and requires standard postoperative care. The ideal dose of ITM for lower extremity arthroplasty under spinal anesthesia is unknown and warrants further investigation.
